# Medical Education and State Examinations for License

**Published:** 1898-06

**Authors:** William Warren Potter

**Affiliations:** President New York State Medical Examining and Licensing Board.


### MEDICAL EDUCATION AND STATE EXAMINATIONS FOR LICENSE.

Conducted by WILLIAM WARREN POTTER, M. D.,
President New York State Medical Examining and Licensing Board.

### EX-CONVICTS CANNOT BE PHYSICIANS.

The United States Supreme Court, April 18, 1898, affirmed the constitutionality of the act of the New York Legislature of 1895 prohibiting persons who have been convicted of and punished for a crime from practising medicine in the state, the opinion being delivered by Justice Brewer. The question arose in the case of a man named Walker, who had served ten years in a penitentiary for an offense committed in 1878, and after his release set up as a physician. The court held that it was within the police power of the state to enact such a law. Justice Harlan delivered a dissenting opinion, saying the law in effect added to the man's punishment and was ex-post facto.

---

### WISCONSIN STATE BOARD.

The regular meeting of the Wisconsin State Board of Medical Examiners (*Wisconsin Medical Recorder*) was held in Milwaukee January 11 and 12, 1898. No candidates for registration were examined but all applicants will be examined at the April meeting. A committee was appointed to examine into the condition and standing of the medical colleges of the state and report to the Board. Several charges of violation of the practice law were ordered investigated. The good effect of the present law is seen in the fact that between seventy-five and eighty registration blanks failed to be returned to the secretary, the probability being that the applicants read the requirements of the board and concluded it would be useless to apply for registration. The greatest number of applicants for registration will be at the July meeting, which follows the commencements of the medical colleges. Future meetings of the board will be held as follows: Milwaukee, the second Tuesday in April; Madison, the second Tuesday in July; Milwaukee, the second Tuesday in October; Oshkosh, the second Tuesday in January, 1899.

---

### OSTEOPATHY IN KENTUCKY.

THE profession of Kentucky (*Western Medical Review*) is to be congratulated on its success in obtaining good legislation. A bill has just passed both houses of the legislature which is an amendment to the practice act now in force, which will make all applicants to practise osteopathy stand an examination before the state board. There was a bitter fight made against the bill by the osteopaths and their friends, but without success. The bill practically prohibits the admission of the followers of Still, for, of course, they cannot pass the required examination.

---

### PUNISHMENT OF A CLERICAL MEDICAL PRACTITIONER.

THE right of a clergyman (*The Texas Medical News*) to attempt to heal by prayer and the laying on of hands has been questioned in New Orleans, where a Catholic priest has been prosecuted for a violation of the State medical-practice law and of a city ordinance relative to clairvoyants, unlicensed practitioners, and the like. The clergyman, while willing to pray over those who desired his services, did not pretend to be able to cure disease, and did not demand any payment for his services, although accepting such small sums as his callers chose to give him. The charge of violating the state law was dismissed, but a fine of twenty-five dollars was imposed for violation of the city ordinance.

---

### KLONDIKE DOCTORS MUST PASS MEDICAL EXAMINING BOARD.

THE *Medical Sentinel*, February, 1898, notifies the numbers of doctors who contemplate trying their fortunes in the Klondike country that it is absolutely necessary for them to pass examination before the Board of Medical Examiners of the Northwest Territory (not British Columbia), whose headquarters is Calgary, of whom Dr. Britt, of Banff, N. W. Territory, is Registrar. The medical law will be enforced by the Provincial authorities. It will save lots of trouble, inconvenience and loss of time for prospecting physicians to read this notice and govern themselves accordingly before they demand nuggets of gold in this country of cold and gold for professional services.—*Virginia Medical Semi-Monthly, Feb. 25, 1898.*

